# The driving mechanism of innovative behavior of vocational education students: an analysis of the role of psychological satisfaction, intrinsic motivation and career needs

**DOI:** 10.3389/fpsyg.2026.1724865

**Published:** 2026-02-18

**Authors:** Xu Xiaoping, Wang Chenhui, Tong Jiawei, Jin Dian, Xiang Fanghe

**Affiliations:** 1 Design College, Jinhua University of Vocational Technology, Jinhua, China; 2 Faculty of Design and Architecture, Putra Malaysia University, Selangor Darul Ehsan, Malaysia; 3 College of Design and Innovation, Zhejiang Normal University, Jinhua, China; 4 Tong Jiawei College of Education, Wenzhou-Kean University, Wenzhou, China; 5 Architectural Engineering College, Jinhua University of Vocational Technology, Jinhua, China

**Keywords:** innovative behavior, intrinsic motivation, psychological satisfaction, self-determination theory, vocational needs

## Abstract

In the context of vocational and technical education, students’ innovative behavior and career development capabilities are considered key outcomes for improving educational quality and social adaptability. Although previous research has pointed out that intrinsic motivation plays a central role in promoting students’ innovative tendencies, its formation and mechanisms remain largely untested. Based on self-determination theory, this study integrates three core psychological variables: basic psychological need satisfaction, intrinsic motivation, and career needs, to construct an integrative path model explaining the innovative behavior of vocational education students. Based on online questionnaire data from 695 students at vocational and technical colleges in China, the study first identified associations among variables using correlation analysis. Chained mediation path regression analysis was then used to examine the mediating mechanisms between the psychological variables. Finally, structural equation modeling was used to verify the overall path model and its fit. The results indicate that basic psychological need satisfaction not only directly enhances students’ intrinsic motivation but also indirectly enhances their perceived need for career development through intrinsic motivation. Career needs play a significant mediating role in the relationship between intrinsic motivation and innovative behavior. This study reveals the multiple psychological mechanisms underlying the innovative behavior of vocational education students and emphasizes that curriculum design should focus on both motivational stimulation and career orientation. This study provides theoretical support and practical implications for building an innovation-oriented vocational education environment.

## 1 Introduction

With rapid changes in labor market demands, vocational education in China is increasingly emphasizing the dual cultivation of technical skills and innovative capabilities (Xu et al., 2023). Innovative behavior, defined as the generation and implementation of new ideas, has become a key competency for vocational college students to address complex career challenges (Fischer et al., 2019). In the field of educational psychology, Self-Determination Theory (SDT) suggests that intrinsic motivation is a key psychological mechanism driving individual innovative behavior (Ryan and Deci, 2000). Previous research has shown that when students’ basic psychological needs—namely, autonomy, competence, and relatedness—are met, their intrinsic motivation is significantly enhanced, leading to greater creative engagement (Ryan and Deci, 2000; Howard et al., 2021; Vallerand, 1997). However, despite the widespread application of SDT in both basic and higher education, systematic research on the relationship between motivation and innovation in vocational education remains relatively scarce. In particular, how individuals are influenced by external career factors within specific educational contexts has not been fully explored (Vallerand, 1997; Ryan et al., 2011).

Why is it important to investigate the link between psychological need satisfaction and innovative behavior among vocational education students? Over the past few years, vocational education has increasingly been recognized as a crucial avenue for cultivating highly skilled workers who can adapt to and thrive in dynamic labor markets (Dai et al., 2022; Kettunen, 2023). As global demand for innovation in the workforce grows, vocational students must not only master technical skills but also develop creative thinking to address future career challenges. However, much of the existing research in vocational education focuses primarily on skills development, while the exploration of how psychological needs satisfaction influences students’ intrinsic motivation and innovation behavior remains underexplored (Conesa et al., 2022; Klaeijsen et al., 2018). Therefore, investigating how the satisfaction of basic psychological needs can activate intrinsic motivation and, in turn, foster innovative behavior offers valuable insights for both theory and practice in vocational education (McClelland and Karabel, 1987). Previous studies have examined educational change, learning environments, and student engagement from psychological and pedagogical perspectives (Orsini et al., 2016; Kritikou and Giovazolias, 2022; Li, 2022).

What are the broader theoretical and practical implications of this research? By constructing an integrated model grounded in SDT, this study aims to systematically examine the pathways linking psychological need satisfaction, intrinsic motivation, and career needs to the prediction of innovative behavior among vocational college students. The study analyses questionnaire data collected from 695 students at vocational and technical colleges in China and employs multiple statistical methods, including correlation analysis, chained mediation path regression, and structural equation modeling (SEM). The findings not only provide new empirical support for the expanded applicability of the SDT framework in vocational education contexts but also offer valuable insights into the psychological mechanisms underlying innovative behavior in vocational students (Kettunen, 2023; Gagné and Deci, 2005).

The significance of this study lies in several key aspects. First, it extends the application of SDT in vocational education by exploring how psychological need satisfaction influences intrinsic motivation and innovative behavior. Second, it fills a gap in the current literature on the relationship between motivation and innovation in vocational education, particularly by examining how career-related factors interact with psychological need satisfaction and intrinsic motivation. This study contributes to the development of more robust theoretical frameworks in educational psychology and provides empirical data for educational reforms (Gagné and Deci, 2005; Kettunen, 2023). Furthermore, the findings of this study offer practical implications for vocational education practice. By highlighting the role of psychological need satisfaction in enhancing intrinsic motivation, this research underscores the importance of designing curricula that promote autonomy, competence, and relatedness among students, thereby fostering a more innovation-oriented learning environment (Ryan et al., 2011; Fischer et al., 2019).

### 1.1 Innovative behavior

Innovative behavior refers to individuals’ proactive engagement in generating, promoting, and implementing new ideas to improve processes or outcomes. In vocational education, innovative behavior is increasingly recognized as a key competency for enhancing students’ problem-solving abilities, technical adaptability, and future employability (Messmann and Mulder, 2011). However, many studies in this area focus on general education settings, and less attention has been paid to vocational education, where students are primarily oriented toward skills application. Therefore, this study fills a gap by exploring innovative behavior in vocational education and emphasizing how it can be fostered within a structured curriculum. In vocational education, students often encounter a more practical, hands-on learning environment. Stimulating their innovative potential within this highly structured curriculum has become a pressing issue for educational reform. A shift from the general focus on classroom engagement to practical, real-world problem-solving contexts is needed to enhance the relevance of innovation in vocational education. Some studies have found that positive student evaluations of the teaching environment, enhanced self-efficacy, and a sense of control over their goals may help stimulate innovative attempts.

Furthermore, factors such as cognitive flexibility, exploratory tendencies, and career planning awareness are also thought to be associated with innovative performance (Devloo et al., 2015; Klaeijsen et al., 2018). While these factors are critical, there remains a lack of empirical research linking them to vocational education’s specific focus on technical and career-oriented skills. However, most current research on student innovation focuses on general higher education students. Vocational education students exhibit significant differences in learning motivation, curriculum structure, and developmental orientation, and targeted empirical research is lacking. Thus, this study expands the scope by directly addressing the vocational context and its unique challenges. Vocational education emphasizes the application of skills and the connection to job roles. Students’ behavior may be driven by their understanding and expectations of their future career paths (Dai et al., 2022). Therefore, further analysis of the internal psychological mechanisms underlying innovative behavior among vocational education students is necessary, particularly the potential interactions among intrinsic motivation, psychological need satisfaction, and career needs. Such analysis is expected to provide theoretical support and practical approaches for systematically cultivating innovative capabilities in applied education contexts.

### 1.2 Self-determination theory: focusing on intrinsic motivation

Self-Determination Theory (SDT) provides a systematic framework for understanding human motivation and its role in learning and innovation. According to SDT, intrinsic motivation arises when individuals engage in activities for their own interest and enjoyment rather than for external pressure or rewards (Ryan and Deci, 2000). A central assumption of SDT is that intrinsic motivation is supported by the satisfaction of three basic psychological needs: autonomy, competence, and relatedness. Autonomy refers to individuals’ sense of volition and control over their actions, competence reflects their perceived ability to master tasks, and relatedness involves feelings of social connection and belonging (Howard et al., 2021; Fischer et al., 2019). According to SDT, intrinsic motivation is based on the fulfillment of three basic psychological needs: autonomy (a sense of personal control over one’s behavior), competence (a belief in one’s ability to complete tasks), and relatedness (a sense of connection and recognition through social relationships). While these needs have been well-established in the context of general education, their specific impact on vocational education—where the emphasis is on career readiness and skill application—has not been fully explored. When educational environments effectively support these basic psychological needs, students are more likely to develop strong, lasting intrinsic motivation, leading to more proactive, innovative behavior. For example, classroom teaching methods that empower students to choose can enhance their autonomy; designing challenging yet manageable tasks can foster a sense of competence; and active interaction between teachers and peers can foster a sense of belonging and social identity (Ryan et al., 2011; Kruse et al., 2024). Conversely, when learning situations restrict students’ autonomy, deny their abilities, or lack emotional support, their intrinsic motivation is suppressed, and their willingness to engage in innovative activities may also be weakened. Although SDT is widely used in general education settings, research in the context of vocational education remains relatively scarce. In particular, in vocational education, students’ psychological motivations are often intertwined with their career orientations and career plans, and a complex interactive relationship may exist between intrinsic and career motivation. However, it is important to distinguish between two related but conceptually different constructs: psychological need satisfaction and vocational needs. Psychological need satisfaction focuses on fulfilling the basic needs of autonomy, competence, and relatedness within the learning environment, whereas vocational needs focus on career development goals that are more extrinsically motivated (Ryan and Deci, 2000). While both constructs influence motivation, they do so in different ways: psychological need satisfaction is linked to engagement in the learning process, while vocational needs are tied to future career aspirations. This study distinguishes these constructs by incorporating career needs as a mediating variable to explore how they interact with intrinsic motivation and psychological need satisfaction. By doing so, we aim to provide a more comprehensive understanding of the psychological drivers of innovative behavior in vocational education students. By distinguishing between intrinsic motivation and career-driven motivation, this study provides a nuanced contribution to SDT, extending its application to vocational contexts and offering new insights into how students’ career expectations interact with their intrinsic motivation to foster innovation.

### 1.3 Integrating SDT and career motivation in vocational education

SDT and career motivation theory address motivation from different but complementary perspectives. SDT focuses on how motivation emerges from the satisfaction of basic psychological needs—autonomy, competence, and relatedness—within a learning environment (Ryan and Deci, 2000; Howard et al., 2021). Career motivation theory, by contrast, emphasizes future-oriented considerations, such as anticipated career roles and external professional requirements, that influence individuals’ effort investment and goal-directed behavior (Kier and Blanchard, 2021; Kettunen, 2023). In vocational education, learning is closely linked to future employment, making it difficult to separate the two perspectives. Psychological need satisfaction functions as a proximal motivational condition that supports students’ intrinsic motivation during learning activities (Ryan and Deci, 2000; Conesa et al., 2022). In this study, career needs refer to students’ subjective perceptions of future occupational expectations and competencies, reflecting how external professional requirements are interpreted and internalized at the individual level. Career needs serve as a more distal motivational influence that shapes the direction of learning-related behavior (Messmann and Mulder, 2011; Dai et al., 2022). From the perspective of SDT, career-related goals are not necessarily external or controlling. When students perceive learning activities as relevant to their future careers, these goals can become partially internalized, strengthening intrinsic motivation and sustained engagement (Ryan et al., 2011; Knox, 2022). Related terms, such as career demands, professional requirements, or career aspirations, are therefore not treated as separate constructs in the present study but are understood as external reference points or internalized expressions that inform students’ career needs. In this sense, intrinsic motivation facilitates the internalization of career needs, linking present learning experiences to future-oriented, innovative behavior. Based on this view, the present study treats SDT and career motivation theory as complementary frameworks. SDT explains how motivation is activated through psychological need satisfaction, while career motivation theory helps explain how this motivation is directed toward career-related innovative outcomes. For conceptual clarity and consistency, career needs are used throughout the manuscript as the core construct representing students’ internalized, career-oriented motivational expectations. Integrating these perspectives provides a more coherent explanation of innovative behavior in vocational education contexts, where learning is inherently both interest-driven and career-oriented (Klaeijsen et al., 2018; Fischer et al., 2019).

### 1.4 Intrinsic motivation and the satisfaction of basic psychological needs in students

Intrinsic motivation plays a central role in the learning process. It stems from an individual’s interest in the learning content and the resulting psychological satisfaction. In educational research, intrinsic motivation is widely considered a key psychological variable predicting student engagement, persistence, and academic performance. Numerous empirical studies have shown a significant positive correlation between intrinsic motivation and academic achievement, learning resilience, and long-term commitment to the subject (Maulana et al., 2013; Schweder and Raufelder, 2024; Stolk et al., 2018). However, while intrinsic motivation is a significant predictor of engagement and achievement, research on how it interacts with specific vocational needs is limited, especially when students’ learning goals are highly career-oriented. This study aims to fill this gap by integrating intrinsic motivation with career-focused aspirations, offering a broader view of how motivation works in vocational education. It can be argued that intrinsic motivation not only influences the quality of the learning process but also, to a certain extent, shapes students’ innovative tendencies and problem-solving abilities. Given the crucial role of motivation in learning and development, clarifying the factors that effectively support or inhibit the formation of intrinsic motivation is of great theoretical and practical significance. SDT states that the development of intrinsic motivation depends on the satisfaction of three basic psychological needs: autonomy, competence, and relatedness. These psychological needs reflect an individual’s sense of control, ability, and social belonging in the learning process (Alivernini et al., 2023; Cerasoli et al., 2016; Chabursky et al., 2024; Wang, 2024). An educational environment that meets these needs is more likely to stimulate students’ spontaneous learning interests and, in turn, encourage them to participate in innovative activities actively. However, despite the widespread recognition of the importance of intrinsic motivation, the existing literature lacks systematic research on students’ subjective perceptions of whether their basic psychological needs are met, and the specific mechanisms by which these perceptions influence intrinsic motivation. Recent research has shifted its focus to the dynamic relationship between intrinsic motivation and psychological need satisfaction. For example, studies have shown that when students perceive that learning tasks provide them with sufficient autonomy and space to develop their abilities, their willingness to participate in project-based or exploratory learning increases significantly. Furthermore, factors such as teacher support, peer interaction, and classroom atmosphere have been found to further enhance students’ learning motivation and innovative performance by satisfying their social connectedness. These findings collectively emphasize the critical role of the educational environment in mobilizing intrinsic motivation, particularly in learning tasks that foster initiative and creativity. Therefore, an in-depth exploration of the relationship between psychological need satisfaction and intrinsic motivation in the context of vocational education is of great value for understanding the drivers of students’ innovative behavior and for optimizing teaching strategies.

### 1.5 Measurement instruments

The psychological need satisfaction scale used in this study was adapted to capture the three core dimensions of SDT: autonomy, competence, and relatedness. For this study, a subset of items was selected from the original scale based on their theoretical relevance to the constructs of intrinsic motivation and innovation behavior. This adjustment helps ensure that the scale is appropriate for the vocational education context and maintains its validity. The rationale for selecting this subset was to ensure that each of the core dimensions of SDT was adequately represented, while also maintaining a manageable scale length to minimize respondent fatigue. In particular, items that best reflected the fundamental aspects of autonomy, competence, and relatedness in the vocational education context were retained. This selection procedure was guided by both theoretical considerations and empirical findings that suggest these dimensions are crucial for fostering intrinsic motivation in educational settings (Ryan and Deci, 2000). The selected items were carefully evaluated for their ability to capture the essence of the constructs without introducing redundancy or significant loss of validity. Furthermore, reliability analyses (e.g., Cronbach’s α) confirmed that the subset of items retained from the original scale demonstrated acceptable internal consistency (α > 0.80) for each construct.

Since innovative behavior, psychological satisfaction, and career needs are all related to intrinsic motivation, we propose the following hypotheses:

Career needs refer to individuals’ cognition and expectations regarding future career goals, role positioning, and external professional requirements informing career needs during their education and career development. These needs typically include considerations such as career path selection, career role clarity, and the accumulation of professional competencies (Li et al., 2024; Wang et al., 2024; Zhang et al., 2019). In educational settings, the satisfaction of career needs is considered a distal driver of students’ intrinsic motivation. When students believe their current learning efforts effectively serve their future career development goals, they are more likely to exhibit higher levels of intrinsic motivation. This mechanism can be explained by the “meaning construction” dimension of self-determination theory: satisfying career-related needs strengthens students’ understanding of the goal value and reality of learning activities, thereby stimulating a stronger willingness to participate autonomously. Empirical research also supports this theoretical approach. For example, Knox (2022) found a significant positive correlation between students’ clarity of their future career roles and their intrinsic motivation levels (Knox, 2022). Similarly, other studies have found that when students anticipate acquiring key career skills through their current education, their learning motivation and proactive participation are significantly enhanced. This relationship is particularly important in the context of vocational education, as this group’s learning goals are generally more career-oriented. Therefore, whether students can see the connection between their learning process and their future career may directly affect their intrinsic motivation level and further innovation investment behavior:

Based on the above theoretical and empirical foundations, the following research hypotheses are proposed:

*H1*: Career needs are positively correlated with students’ intrinsic motivation.

In educational psychology, intrinsic motivation is widely regarded as a core psychological mechanism that drives students’ deep learning, persistence, and adaptive learning behaviors. Intrinsic motivation refers to engagement in learning activities driven by inherent interest and enjoyment, rather than by external rewards or pressures (Howard et al., 2021; Vasconcellos et al., 2020). A substantial body of research has consistently shown that higher levels of intrinsic motivation are associated with better learning outcomes, stronger persistence, and deeper engagement in academic tasks (Zeng et al., 2024). According to SDT, the development and maintenance of intrinsic motivation depend largely on the satisfaction of three basic psychological needs: autonomy, competence, and relatedness. Psychological need satisfaction refers to the extent to which individuals experience choice and volition in learning activities (autonomy), feel capable of mastering task challenges (competence), and perceive support and recognition from significant others (relatedness) (Vergara-Morales and Del Valle, 2021; Zheng et al., 2023). When these needs are satisfied, individuals are more likely to internalize learning activities and experience intrinsic motivation. Empirical studies provide strong support for this theoretical proposition. For instance, Vallerand et al. (2008) demonstrated that students’ satisfaction with autonomy, competence, and relatedness significantly predicted their intrinsic motivation in academic contexts. More recent research has further shown that supportive teaching practices, positive classroom climates, and teacher-student interactions can enhance students’ psychological need satisfaction, thereby indirectly strengthening their intrinsic motivation (Conesa et al., 2022; Tian and Shen, 2023). These findings suggest that psychological need satisfaction functions as a critical antecedent of intrinsic motivation, particularly in learning environments that emphasize autonomy, exploration, and active engagement.

Based on self-determination theory and existing empirical evidence, the following hypothesis is proposed:

*H2*: Psychological need satisfaction is positively related to students’ intrinsic motivation.

Intrinsic motivation is widely considered one of the key psychological variables driving students’ innovative behavior. It generally refers to students’ active participation in tasks driven by inherent interest, curiosity, or intrinsic satisfaction, rather than reliance on external rewards or social pressure. In educational psychology research, intrinsic motivation is not only significantly correlated with students’ academic performance but also positively influences their creative thinking and innovative behavior. In the learning process, innovative behavior primarily manifests as students’ ability to generate new ideas, solve complex problems, or experiment with novel approaches. This behavior reflects an individual’s ability to proactively mobilize creative resources to engage in cognitive restructuring and strategic adjustment when faced with challenging tasks. In educational settings, intrinsic motivation is considered the core driving force behind students’ creative thinking and practical exploration. When students identify with and are interested in the learning content and have a degree of autonomy in their learning, they are more likely to proactively explore, iterate their thinking, and generate innovative solutions. Numerous empirical studies further support this relationship. For example, Hennessey and Amabile (2010) found that intrinsic motivation significantly predicted student creativity across various disciplines. In particular, when learning tasks are challenging and students perceive sufficient freedom of choice, their creative engagement is higher. Furthermore, research has found that students with high intrinsic motivation are more likely to actively seek novel strategies in collaborative and interdisciplinary learning environments, demonstrating stronger innovation capabilities. Given the practice-oriented nature of innovative behavior in vocational education, understanding the impact of intrinsic motivation on students’ innovative behavior is of practical significance for fostering their technological adaptability and problem-solving abilities. Therefore, based on the aforementioned theoretical foundation and research evidence, this study proposes the following hypotheses:

*H3*: Intrinsic motivation is significantly positively correlated with students’ innovative behavior.

According to Maslow’s Hierarchy of Needs, individuals must first satisfy their basic psychological needs, such as autonomy, competence, and social belonging, before higher-order needs (such as self-actualization) can be activated. This theory emphasizes that when individuals’ psychological needs are satisfied, they are motivated to actively pursue higher-order goals, including achieving personal values and career aspirations. Furthermore, Person-Job Fit Theory suggests that individuals’ perceptions and choices of career roles are largely determined by their judgment of the degree of fit between their needs and external job characteristics. When individuals experience intrinsic psychological satisfaction, they are more likely to actively seek career paths that align with their interests, abilities, and values, and demonstrate greater engagement and goal clarity in career planning and skills development (Jemini-Gashi and Hoxha, 2024; Kuzborska et al., 2024; Picker-Roesch and Lang, 2024; Yu et al., 2018). Related research supports this theoretical mechanism. For example, empirical evidence shows that positive experiences of autonomy and competence during education enhance identification with and willingness to participate in future career roles. Furthermore, emotional satisfaction in learning situations can also help strengthen students’ intrinsic motivation for career development, thereby influencing their perceived intensity of career needs and their chosen career path. This relationship is particularly important in the context of vocational education. Students’ learning experiences not only shape their current motivational structures but also directly relate to their expectations and actions regarding future career paths. Therefore, exploring whether psychological satisfaction enhances students’ sensitivity and responsiveness to career needs can help understand how motivational mechanisms function in innovation-oriented career development.

Based on the theoretical and empirical foundations described above, the following research hypotheses are proposed:

*H4*: Satisfaction (i.e., the satisfaction of basic psychological needs) is significantly positively correlated with students’ perceived career needs.

With the ongoing transformation of industrial structures and the increasing demand for job skills, future career development demands higher levels of adaptability and problem-solving skills from students. In this context, students must not only master professional knowledge but also possess the ability to innovate and proactively change continuously. The pressure to meet goals and the challenges posed by career development orientations often prompt students to focus on proposing improvement plans, experimenting with new approaches, and driving practical change during their learning phase, thereby enhancing their adaptability and competitiveness in future work situations. From a motivational perspective, career needs reflect not only students’ cognition of future career goals but also their perceived resources and action tendencies to achieve them. Specifically, when students perceive that future career success requires innovative abilities, they are more likely to exhibit exploratory behavior and creative thinking in their current learning activities. This internalized career drive serves as a psychological mechanism that combines external incentives with internal motivation, thereby promoting innovative behavior. Existing research provides both theoretical and empirical support. For example, Bulman et al. (2022) noted that the clearer an individual’s understanding of career development, the greater their proactive and creative behavior during their learning phase. Chao et al. (2009) and Miklós-Thal and Ullrich (2016) further emphasize that specific types of career incentives, such as recognition of the value of technological innovation and expectations of enhanced job competencies, significantly enhance students’ innovative engagement during their studies (Bulman et al., 2022; Chao et al., 2009; Miklós-Thal and Ullrich, 2016). In vocational education settings, students’ perception of career needs is particularly pronounced, as course content is highly relevant to job roles. Therefore, understanding how career needs stimulate students’ innovative behavior can help develop more practical mechanisms for cultivating innovative capabilities.

Based on the above theoretical analysis and empirical evidence, the following research hypothesis is proposed:

*H5*: There is a significant positive correlation between students’ perceived career needs and their innovative behavior.

**FIGURE 1:**
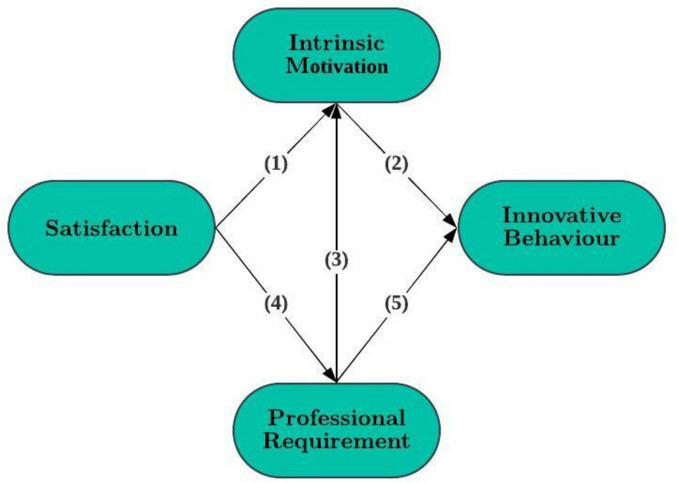
Conceptual model with the accompanying hypotheses in brackets.

This study aims to expand the theoretical understanding of student innovative behavior and to explore the multiple psychological mechanisms underlying it and their potential interactions. Specifically, the study focuses on how individual innovative behavior in a learning environment is jointly influenced by factors such as basic psychological need satisfaction, career-related motivation, and intrinsic motivation. By identifying these key psychological drivers, the study aims to provide theoretical and empirical support for promoting student innovative behavior in educational practice. The core question explored is: How do students’ psychological satisfaction, perceived career needs, and intrinsic motivation influence their innovative behavior during their time in school? To systematically address this question, this paper constructs a theoretical model (see Figure 1) based on self-determination theory and relevant empirical findings. The model hypothesizes that three variables—two basic psychological needs (autonomy and competence) and one career-oriented extrinsic motivation (career needs)—foster students’ intrinsic motivation, thereby further promoting their innovative behavior. The proposed structural model not only integrates the aforementioned theoretical approaches but also provides an operational framework for testing five specific hypotheses. Given that the hypothesized model consists of only four core variables, relevant control variables (e.g., gender, age, academic year, and major) will be incorporated in the model testing to strengthen the robustness of the findings. An empirical analysis of the model’s path relationships will further verify the positive relationships among the variables, thereby deepening our understanding of the psychological mechanisms underlying students’ innovative behavior. In addition, correlation analyses among the study variables will be reported to support the proposed relationships. Furthermore, evidence of discriminant validity will be provided to ensure the constructs are distinct and accurately measured. To address concerns regarding the reliance on self-reported data, appropriate tests for common method bias (e.g., Harman’s single-factor test) will be conducted and reported in the analysis. These steps will enhance the reliability and validity of the findings, ensuring a more comprehensive understanding of the factors influencing students’ innovative behavior.

## 2 Materials and methods

### 2.1 Data collection

Data were collected using a structured online questionnaire administered to students enrolled in a vocational and technical college in Zhejiang Province. The data collection period lasted 15 days and concluded on November 12, 2024. A total of 695 valid questionnaires were obtained after excluding incomplete or invalid responses. The internal consistency reliability of the overall questionnaire was high (Cronbach’s α = 0.95). The final sample consisted of 407 male students (58.6%) and 288 female students (41.4%). Participants ranged in age from 16 to 22 years, with a mean age of 19.1 years. Participation was voluntary, and all respondents were informed of the research purpose, data usage, and confidentiality prior to completing the survey. Electronic informed consent was obtained from all participants to ensure ethical compliance. All measurement instruments were adapted from established and validated scales used in prior research. Minor wording adjustments were made to ensure clarity and relevance to the vocational education context. All items were rated on a 7-point Likert scale ranging from 1 (“strongly disagree”) to 7 (“strongly agree”).

#### 2.1.1 Innovative behavior

The original scale consists of 20 items assessing idea generation, idea promotion, and idea implementation. Based on the educational context and research objectives, six representative items were selected to assess students’ innovative behavior in learning and in practical activities. Sample items include “I look for new ways to improve my current learning methods” and “I proactively propose new ideas for learning tasks.” Higher scores indicate a higher level of innovative behavior.

#### 2.1.2 Intrinsic motivation

Intrinsic motivation was assessed using items adapted from the Intrinsic Motivation Inventory developed by Deci and Ryan, grounded in self-determination theory. The original scale includes 45 items measuring interest, enjoyment, and perceived competence. To reduce respondent burden, five items most relevant to learning motivation were selected. Sample items include “I enjoyed engaging in this learning task” and “I felt confident in completing this task.” Higher scores reflect higher levels of intrinsic motivation.

#### 2.1.3 Career needs

Students’ career needs were measured using adapted items from the Copenhagen Psychosocial Questionnaire (COPSOQ), specifically the job demands and job content dimensions. The items were modified to reflect students’ perceptions of future occupational expectations, task complexity, and skill requirements. Sample items include “Future jobs will require continuous skill development” and “I expect future work tasks to be highly complex.” Higher scores indicate stronger perceived career needs.

#### 2.1.4 Psychological need satisfaction

Psychological need satisfaction was measured using selected items from the Basic Psychological Need Satisfaction Scale for Students, based on self-determination theory. The scale assesses satisfaction of autonomy, competence, and relatedness in learning contexts. To ensure parsimony, five items were selected to represent these three dimensions. Sample items include “I have freedom in choosing how I learn” (autonomy), “I feel capable of handling my learning tasks” (competence), and “I receive support and recognition from teachers and peers” (relatedness). Higher scores indicate higher levels of psychological need satisfaction.

### 2.2 Data analysis

Three complementary statistical techniques were used to examine the relationships among psychological need satisfaction, intrinsic motivation, career needs, and innovative behavior: correlation analysis, serial mediation regression analysis, and SEM. These methods were applied sequentially to assess bivariate associations, indirect effects, and the overall model structure.

#### 2.2.1 Correlation analysis

Pearson’s correlation coefficients were calculated to examine the strength and direction of associations among the key variables. This analysis provided preliminary evidence regarding whether the variables were significantly related and suitable for subsequent mediation and structural modeling. Correlation coefficients and significance levels were reported for all study variables.

#### 2.2.2 Chain mediation path regression analysis

To examine how basic psychological needs influence innovative behavior through intrinsic motivation and career needs, we employed serial mediation regression analysis. This method allows exploration of indirect effects, revealing complex, multi-step pathways through which the independent variable affects the dependent variable via mediating variables.

Procedure: A serial mediation model was constructed with psychological need satisfaction as the independent variable (X), intrinsic motivation (M1) and career needs (M2) as mediators, and innovative behavior as the dependent variable (Y). The following paths were tested:

(1) Psychological needs satisfaction → intrinsic motivation → innovative behavior;
(2) Psychological needs satisfaction → career needs → innovative behavior;
(3) Psychological needs satisfaction → intrinsic motivation → career needs → innovative behavior (chain mediation path);

To estimate path coefficients and test the significance of mediation effects, this study employed partial least squares (PLS) path modeling, combined with bootstrap resampling (*n* = 5,000) to estimate confidence intervals. The significance of the indirect effect was determined as follows: if the 95% confidence interval (CI) did not contain 0, the mediation effect was considered significant. Furthermore, standardized regression coefficients (β) and *p*-values for the direct effect (c’ path), the total effect (c path), and each mediation path were reported to comprehensively assess the strength of the mediation mechanism and path structure. All data analyses were conducted in SPSS PRO to ensure statistical robustness and transparency of interpretation. To control for potential confounding variables, gender, grade, and major type were included as control variables, and covariates were adjusted in the model. Using chained mediation regression analysis, this study aimed to reveal how basic psychological needs, via the sequential transmission of intrinsic motivation and career orientation, gradually drive students’ innovative behavior, thereby providing more systematic empirical support for the motivation-behavior mechanism in the context of vocational education.

#### 2.2.3 Structural equation modeling

SEM was used to test the overall fit of the proposed theoretical model and to examine direct and indirect relationships among latent variables. Psychological need satisfaction, intrinsic motivation, and career needs were specified as exogenous latent variables, with innovative behavior specified as the endogenous latent variable. Model fit was evaluated using multiple indices, including χ^2^/df, RMSEA, CFI, and TLI. Standardized path coefficients were estimated to assess the strength and significance of hypothesized relationships. SEM analyses were conducted to provide a comprehensive test of the measurement and structural components of the model.

## 3 Results

### 3.1 Descriptive statistics

Table 1 presents the descriptive statistics for each structural component of the hypothesized model. The results show that respondents scored highest on the “intrinsic motivation” component (*M* = 6.01, *SD* = 0.96), while those on the “job demands” component scored lowest (*M* = 4.97, *SD* = 0.77). The relationships among the theoretical constructs were examined using Pearson’s correlation analysis. The results indicated that all indicators were significantly correlated (*p* < 0.01), supporting the hypothesized relationships among the variables.

**TABLE 1:** Means (*M*) and standard deviations (*SD*) of the constructs studied.

Construct	*M*	*SD*
Satisfaction	5.69	0.79
Intrinsic motivation	6.01	0.96
Innovative behavior	5.47	0.82
Profession requirements	4.97	0.77

### 3.2 Correlation results

**FIGURE 2:**
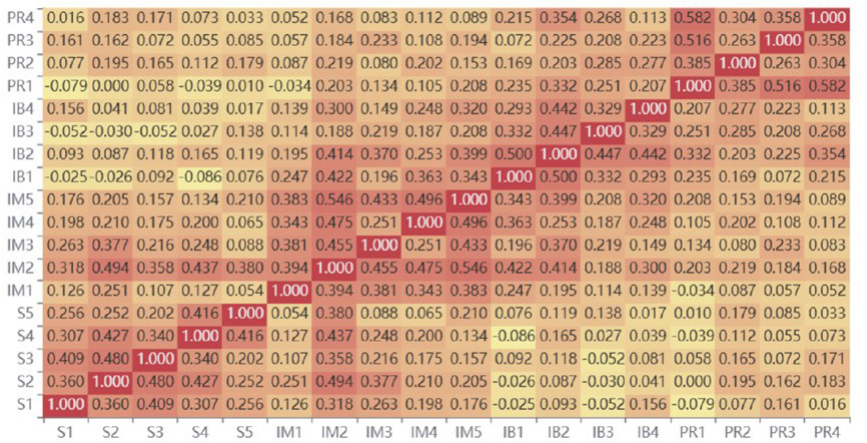
Pearson correlation heatmap between measurement items. Darker colors represent stronger correlations, with red indicating positive correlations and yellow indicating negative correlations. Correlation analysis based on standardized values (*N* = 695).

Before conducting path modeling, Pearson correlation analyses were performed on the measurement items for each latent variable in this study. The results are shown in Figure 2. The heat map clearly demonstrates the degree of linear correlation between the variables, with the color depth indicating the strength and direction of the correlation coefficient. The results show that the correlations between the basic psychological needs satisfaction (S1–S5) items are high (e.g., *r* = 0.36 between S1 and S2, *r* = 0.41 between S1 and S3), indicating good internal structural consistency of the scale. The internal correlations of the intrinsic motivation scale (IM1ds satisfactionmoderate to high (e.g., *r* = 0.38 between IM2 and IM5, *r* = 0.43 between IM3 and IM5), supporting its convergent validity as a latent variable. In terms of cross-dimensional correlations, psychological satisfaction and intrinsic motivation items generally show positive correlations, especially between S3 and IM2 (*r* = 0.48) and between S2 and IM2 (*r* = 0.48), suggesting that psychological need satisfaction may positively affect intrinsic motivation. Furthermore, there were moderate positive correlations between intrinsic motivation and innovative behavior items (IB1e moderate p*r* = 0.50 between IM5 and IB2 and *r* = 0.47 between IM4 and IB2, providing preliminary support for their proposed mediation pathways in the chain model. The correlations between the career needs dimension (PR1–PR4) and other variables were generally low. With the exception of PR3 and S4 (*r* = 0.43) and PR3 and IM2 (*r* = 0.49), most coefficients ranged from 0.1 to 0.2, potentially reflecting the independence of career needs as an extrinsic motivation variable.

### 3.3 Regression analysis results of chain mediation paths

To further examine the mechanism by which psychological satisfaction influences students’ innovative behavior, this study constructed three sets of multivariate linear regression models to explore whether it exerts an indirect effect through intrinsic motivation. This analysis aims to provide preliminary empirical support for the mediation path in the subsequent structural equation model. As shown in Table 2, in Model 1, the direct predictive effect of psychological satisfaction on innovative behavior is weak (β = 0.043, *R*^2^ = 0.001), indicating that its effect alone is limited. In Model 2, by adding intrinsic motivation to this model, the explanatory power of the model is significantly enhanced (*R*^2^ = 0.127). Intrinsic motivation has a strong, significant predictive effect on innovative behavior (β = 0.347, *p* < 0.001), while the direct effect of psychological satisfaction shifts from positive to negative (β = –0.086), suggesting the possibility of a complete mediation mechanism. Furthermore, Model 3 shows that psychological satisfaction has a significant positive predictive effect on intrinsic motivation (β = 0.372, *R*^2^ = 0.097, *p* < 0.001), supporting the premise of the mediation path.

**TABLE 2:** Summary of regression models for chain mediation paths.

Model	Dependent variable independent	Variable	β (standardized)	*R* ^2^
M1	Innovative behavior	psychological satisfaction	0.043	0.001
M2	Innovative behavior	psychological satisfaction, intrinsic motivation	−0.086, 0.347	0.127
M3	Intrinsic motivation	psychological satisfaction	0.372	0.097

**FIGURE 3:**
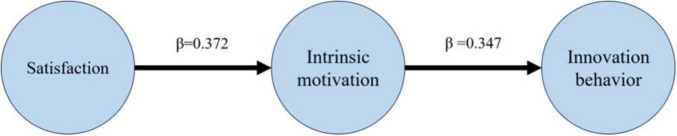
Chain intermediary path structure diagram.

Figure 3 visualizes the structure of this chain mediation pathway. Standardized path coefficients indicate that psychological satisfaction positively predicts intrinsic motivation, which in turn positively predicts innovative behavior. However, after controlling for the mediating variables, the direct effect of psychological satisfaction on innovative behavior becomes negative. This pattern suggests that the psychological satisfaction individuals gain in learning situations may indirectly promote their engagement in innovative behavior by stimulating their intrinsic motivation.

### 3.4 Structural equation model results

Table 3 reports the key fit indices for the SEM, including the comparative fit index (CFI), goodness of fit index (GFI), degrees of freedom ratio (χ^2^/df), chi-square statistic (χ^2^), root mean square error of approximation (RMSEA), and the *p*-value for the model significance test. These indices were used to assess the overall fit of the model and determine how well the hypothesized model represented the data.

**TABLE 3:** Results of model parameters.

Criteria	Result	Threshold			Evaluation
		Terrible	Acceptable	Excellent	
CFI	0.961	< 0.9	> 0.9	> 0.95	Accept
GFI	0.947	< 0.9	> 0.9	> 0.95	Accept
DF	695				Accept
Chi square	1,070	> 5			Accept
RMES	0.052	0.08 <	< 0.08	< 0.06	Accept
*p*-value	0.033	0.05 <	< 0.05	< 0.01	Accept

**FIGURE 4:**
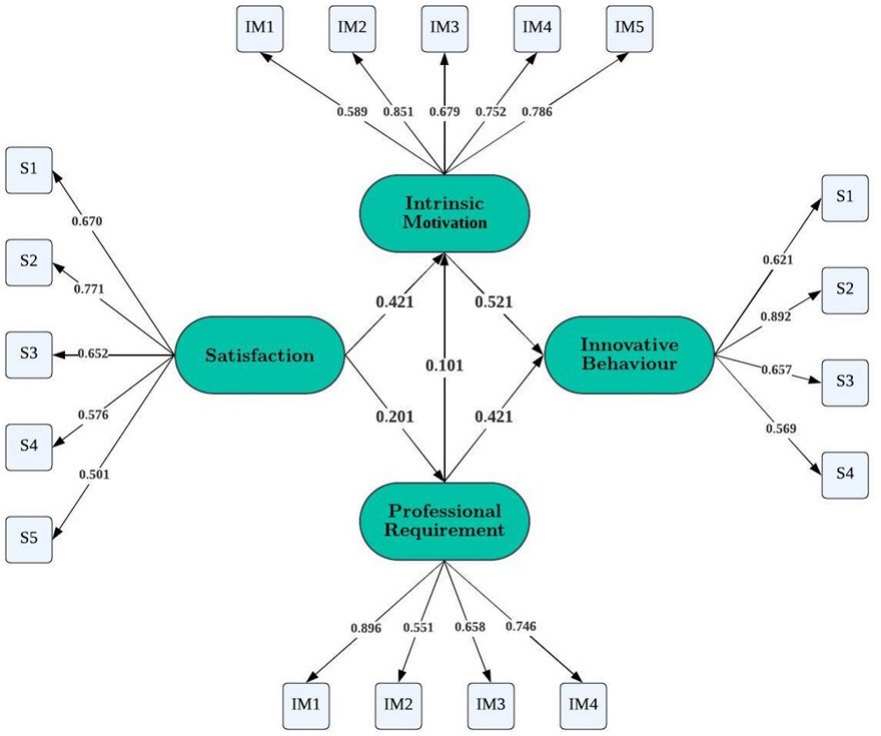
Final structural model representing the standardized relationships. All path coefficients are standardized estimates (*N* = 695). *P*< 0.05 indicates statistical significance. The figure shows the path relationship between psychological satisfaction, career needs, intrinsic motivation and students’ innovative behavior.

The results indicate that the model demonstrated a good fit, with all key fit indices meeting the recommended thresholds for model evaluation. Specifically, the Comparative Fit Index (CFI) was 0.961 and the Goodness-of-Fit Index (GFI) was 0.947, both exceeding the 0.90 threshold, indicating that the model fits the data well. Additionally, the Root Mean Square Error of Approximation (RMSEA) was 0.052, within the acceptable range of < 0.08, suggesting minimal model error and a good representation of the data. Furthermore, the Chi-square statistic (χ^2^) was 1,070, with a *p*-value of 0.033. This *p*-value indicates that the model fits the data significantly better than the unstructured model (null hypothesis), as it is below the 0.05 threshold commonly used to determine model significance (Figure 4). These results confirm that the hypothesized structural equation model provides a satisfactory fit, supporting the proposed relationships. The good model fit, combined with the significant *p*-value for the chi-square test, suggests that the model adequately represents the relationships among the key variables.

### 3.5 Reliability and validity of constructs

In addition to examining model fit, the study assessed the reliability and validity of the constructs used. To evaluate the internal consistency of the measurement model, we calculated Cronbach’s alpha, composite reliability (CR), and average variance extracted (AVE) for each latent variable. The Cronbach’s alpha values for all constructs exceeded the recommended threshold of 0.70, indicating strong internal consistency. Specifically, psychological satisfaction had an alpha of 0.85, intrinsic motivation of 0.88, career needs of 0.82, and innovative behavior of 0.87. These values indicate that the measurement scales used in the study are reliable. To further confirm the reliability of the constructs, composite reliability (CR) values were calculated; all exceeded the 0.70 threshold, indicating satisfactory reliability. The CR values for psychological satisfaction, intrinsic motivation, career needs, and innovative behavior were 0.90, 0.91, 0.88, and 0.89, respectively. Additionally, average variance extracted (AVE) values were also computed to assess convergent validity. The AVE values for all constructs were above the 0.50 threshold, with psychological satisfaction at 0.72, intrinsic motivation at 0.76, career needs at 0.70, and innovative behavior at 0.74. These results indicate that the constructs exhibit good convergent validity, confirming that the measurement model reliably captures the intended variables.

### 3.6 Path coefficients and hypothesis testing

The results of the structural equation model provided support for all the proposed hypotheses. Hypothesis 1 suggested that career needs would significantly and positively predict intrinsic motivation, and this relationship was confirmed (β = 0.101, *p* < 0.05). Hypothesis 2, which posited that psychological satisfaction would predict intrinsic motivation, was also supported (β = 0.421, *p* < 0.01), indicating that satisfaction with autonomy, competence, and relatedness is a stronger predictor of intrinsic motivation than career needs. The central role of intrinsic motivation in predicting innovative behavior was confirmed by Hypothesis 3 (β = 0.521, *p* < 0.01). Given that all variables were standardized, this coefficient indicates that a one standard deviation increase in intrinsic motivation is associated with an increase of approximately 0.52 standard deviations in innovative behavior, suggesting a substantial practical effect. Hypothesis 4 proposed that psychological satisfaction would positively influence career needs, and this relationship was also supported (β = 0.201, *p* < 0.05). Although the magnitude of this effect was relatively modest, it suggests that positive learning experiences contribute to students’ career-related awareness. Finally, Hypothesis 5 suggested that career needs would positively predict innovative behavior, which was confirmed (β = 0.412, *p* < 0.01). In practical terms, a one-standard-deviation increase in perceived career needs corresponds to approximately 0.41 standard deviations in innovative behavior, indicating a meaningful effect size. Overall, these results show that psychological satisfaction primarily influences innovative behavior through indirect pathways, with intrinsic motivation and career needs accounting for a substantial proportion of the variance in students’ innovative behavior.

## 4 Discussion

In today’s knowledge-intensive and rapidly evolving society, vocational education students face unprecedented learning challenges and career transition pressures. This dynamic environment demands not only solid professional skills and theoretical knowledge but also a sustained sense of innovation and practical skills to adapt to the complex demands of technological change and job changes. Within this context, students’ innovative behavior not only shapes their career choices but also plays a key role in improving the quality of vocational education and fostering societal innovation. “Innovation is a key response mechanism for students facing educational and career challenges. Our findings underscore its significance in vocational education, aligning with the view that fostering innovative behavior is critical for developing talent and improving social competitiveness (Birds, 2015; Commeiras et al., 2013; Xu et al., 2023). This supports the theoretical premise that innovation in vocational education should be viewed not merely as an outcome, but as an ongoing, dynamic process driven by students’ internal motivations and external career needs.” Our results further demonstrate that intrinsic motivation, satisfaction of basic psychological needs, and career needs all play significant roles in predicting students’ innovative behavior. Specifically, intrinsic motivation and career needs have a direct, significant positive impact on students’ innovative behavior, consistent with existing research (DeLuca et al., 2024). However, it is important to note that the cross-sectional design of this study prevents us from making definitive causal inferences regarding these relationships. Although career needs are presented as a strong predictor of innovative behavior, further research using longitudinal or experimental designs would be necessary to establish causality. Intrinsic motivation, as the core mechanism driving individual spontaneous learning, strengthens students’ proactive exploration and creative engagement in learning activities. Career needs, on the other hand, represent students’ cognitive expectations of future career goals and tasks. Their intensity significantly increases students’ perception of innovative behavior as a key strategy for achieving career value. While this study suggests a positive relationship between career needs and innovative behavior, we caution that this relationship is based on correlational data, and future research should aim to clarify the directionality of this association. In contrast, while psychological satisfaction did not directly and significantly predict innovative behavior, its effect is more likely to be indirectly mediated through intrinsic motivation. This finding supports the motivational activation pathway in self-determination theory, which states that satisfying basic psychological needs stimulates intrinsic motivation, which in turn promotes the expression of innovative behavior. Again, this relationship should be interpreted with caution, as we cannot infer causal pathways from cross-sectional data alone. Notably, the direct effect of career needs on innovative behavior in this study was stronger than the indirect effect of psychological satisfaction, suggesting the importance of external career-oriented motivation in the context of vocational education (Dai et al., 2022; Li et al., 2023). While we observe this significant association, the cross-sectional nature of the data means we cannot definitively establish that career needs directly cause innovative behavior. Future research should aim to investigate these potential causal pathways. Overall, this study’s findings not only validate the applicability of psychological motivation theory in the vocational education context but also highlight the key role of career-oriented motivation in explaining students’ innovative behavior. Psychological need satisfaction and career needs do not operate in isolation; rather, they jointly influence individuals’ intrinsic motivation, thereby stimulating students’ creative engagement at the behavioral level (Kettunen, 2023). However, these relationships should be considered with the understanding that causality cannot be firmly established through the current study design. This integrated perspective has important theoretical and practical implications for developing more forward-looking vocational education teaching strategies and strengthening the cultivation of innovative talents. From a practical perspective, the magnitude of the observed effects suggests that motivational factors are not only statistically relevant but also meaningful in educational settings. The results indicate that relatively moderate improvements in students’ intrinsic motivation are associated with noticeable increases in innovative behavior. This implies that pedagogical interventions aimed at enhancing intrinsic motivationcreases in tonomy-supportive teaching practices or competence-oriented learning tasks—may lead to observable changes in students’ innovative engagement rather than marginal effects. Similarly, the practical influence of career needs highlights the importance of aligning learning activities with students’ future occupational expectations, suggesting that strengthening the perceived relevance of learning to career development can meaningfully promote innovative behavior in vocational education contexts.

### 4.1 Basic psychological needs and intrinsic motivation

From the perspective of SDT, the satisfaction of basic psychological needs plays a central role in stimulating and maintaining an individual’s intrinsic motivation. Intrinsic motivation refers to individuals engaging in activities driven by interest, enjoyment, or inherent satisfaction, rather than for external rewards or to avoid punishment. According to the fundamental assumptions of SDT, an individual’s intrinsic motivation is highly dependent on the perceived satisfaction of autonomy, competence, and relatedness in a specific context. The findings of this study further confirm this theoretical mechanism.

#### 4.1.1 The role of psychological needs in intrinsic motivation

Students who experience a high degree of autonomous control (e.g., the ability to choose their learning methods), a sense of competence (e.g., a sense of accomplishment in academic tasks), and social connectedness (e.g., emotional support from peers or teachers) during their learning process significantly enhance their intrinsic motivation and, consequently, become more actively involved in innovative behaviors (Ryan and Deci, 2000). This study offers new insights into SDT by extending its applicability to the context of vocational education. While SDT has been widely used in general education, our study provides evidence that psychological satisfaction—specifically autonomy, competence, and relatedness—is an important predictor of intrinsic motivation and innovative behavior in vocational education settings.

#### 4.1.2 SDT in the context of vocational education

These findings contribute to the ongoing development of SDT by demonstrating how it can be applied in a context that emphasizes practical skills and career-oriented learning, a domain that has not been thoroughly explored in previous SDT research. Importantly, this study identifies the mediating role of intrinsic motivation in this process, shedding light on how psychological satisfaction drives students’ innovative behavior by influencing intrinsic motivation. Moreover, this study highlights several conditions under which SDT’s predictions are particularly relevant. For instance, the context of vocational education—which typically involves a more practical, career-focused curriculum—provides a unique setting in which students’ psychological satisfaction plays a crucial role in shaping their motivation and innovative behavior. Unlike traditional educational settings, where intrinsic motivation may be driven more by academic achievement and intellectual exploration, vocational education environments emphasize practical problem-solving and skill development. Our findings suggest that in such contexts, intrinsic motivation may be a more robust driver of creativity and innovation, as students are encouraged to apply their knowledge to real-world challenges.

#### 4.1.3 Theoretical contributions and integration of other theories

This extension of SDT offers new theoretical insights into how psychological satisfaction can foster intrinsic motivation and innovation in career-focused educational environments, providing a deeper understanding of the mechanisms that drive student engagement and creativity. This extension also highlights that SDT can be further refined and contextualized, suggesting that environments with a strong emphasis on practical, career-related skills may offer unique opportunities to engage students’ intrinsic motivation in ways distinct from traditional academic settings. Moreover, integrating Maslow’s Hierarchy of Needs and Person-Job Fit Theory into our model provides a broader framework for understanding how psychological satisfaction in vocational education contexts aligns with students’ career-oriented goals. While SDT primarily focuses on the fulfillment of autonomy, competence, and relatedness in educational settings, Maslow’s theory emphasizes self-actualization that occurs when individuals satisfy higher-order needs, such as personal growth and fulfillment. This concept resonates with SDT’s view of intrinsic motivation, particularly in environments that demand both personal development and practical application, like vocational education. Similarly, the Person-Job Fit Theory offers a critical perspective by emphasizing the importance of aligning students’ perceived career needs with their intrinsic motivation. Our study demonstrates that when students perceive that their career goals align with their educational experiences, they are more intrinsically motivated, leading to greater innovation and engagement in their learning.

#### 4.1.4 Implications for vocational institutions and teachers

The findings of this study suggest several concrete practices that vocational institutions and teachers can adopt to promote students’ innovative behavior by enhancing intrinsic motivation, aligning career needs with learning, and improving psychological satisfaction. At the curriculum level, vocational institutions can reform their curricula to better support students’ intrinsic motivation. Prior research has shown that autonomy-supportive and project-based learning environments are effective in fostering students’ engagement and innovative behavior in vocational and professional education contexts (Messmann and Mulder, 2011; Klaeijsen et al., 2018). Rather than focusing solely on skill transmission, curricula can be organized around authentic problems and open-ended projects that allow students to make meaningful choices regarding topics, tools, or application contexts. In addition, assessment practices can be adjusted to value creativity, problem-solving processes, and iterative improvement, which has been shown to support intrinsic motivation and sustained learning engagement (Ryan and Deci, 2000; Maddens et al., 2023). At the organizational and instructional level, mentorship programs can be introduced to better align students’ career needs with their learning experiences. Career development research suggests that mentoring relationships with teachers, industry professionals, or senior peers help students clarify career goals and understand how current learning activities connect to future occupational roles (Kier and Blanchard, 2021; Kettunen, 2023). In vocational education settings, such mentorship arrangements can support the internalization of career-related goals by providing students with concrete feedback on professional expectations and innovation-related competencies, thereby strengthening the motivational link between learning and career development (Khampirat, 2021). In addition, vocational institutions can implement targeted psychological and educational interventions to enhance students’ psychological satisfaction. Studies grounded in self-determination theory indicate that interventions focusing on autonomy support, competence development, and social connectedness—such as reflective workshops, goal-setting activities, and collaborative learning structures—can effectively enhance students’ psychological need satisfaction and intrinsic motivation (Ryan et al., 2011; Conesa et al., 2022). From a career development psychology perspective, emotional and self-regulatory capacities also play an important role in helping students connect learning experiences with future career goals (Tripon, 2022). Teachers can further promote psychological satisfaction in everyday teaching by providing supportive feedback, encouraging cooperation, and fostering a classroom climate that values exploration rather than error avoidance. Taken together, these findings suggest that fostering innovation in vocational education requires coordinated efforts at the curriculum, instructional, and psychological levels. By combining curriculum reform, mentorship support, and psychologically informed teaching practices, vocational education institutions can create learning environments that not only transmit professional skills but also actively cultivate students’ motivation and innovative capacity.

### 4.2 Career needs and innovative behavior

This study highlights the important role of career needs in predicting innovative behavior among vocational college students. Career needs reflect students’ subjective perceptions of future occupational roles, development pathways, and professional expectations, which shape their learning priorities and behavioral choices during vocational training (Kier and Blanchard, 2021). Specifically, career needs encompass not only the need to master professional skills but also expectations of professional achievement, social status, and role value. These expectations drive students to proactively pursue capacity-building and breakthrough innovations to better cope with future career challenges and changes. The results indicate that career needs have a significant positive impact on students’ innovative behavior (Kirk et al., 2024; Shin and Lee, 2017). Importantly, career needs in this study are not conceptualized as purely external demands. Instead, they function as an internalized motivational orientation that guides students’ learning behavior. When career-related expectations are personally meaningful, students are more likely to pursue innovative approaches to enhance their professional competitiveness and prepare for future work roles (Avsec and Savec, 2021; Thurlings et al., 2015). From a career development psychology perspective, this motivational function of career needs is closely linked to students’ internal psychological resources. Prior research has shown that career-related psychological factors, such as emotional awareness, self-regulation, and perceived career relevance, play an important role in shaping students’ engagement in innovation-oriented learning pathways (Tripon, 2022). Although Tripon (2022) focused on STEM career choice, the findings suggest that when students can emotionally connect their learning experiences with future career goals, they are more likely to engage in proactive exploration and innovative behavior. This perspective supports the view that innovative behavior in vocational education is embedded not only in skill acquisition but also in students’ broader career development processes, reinforcing the link between career needs and innovation identified in the present study. In practice, this drive often manifests itself in students actively participating in project-based learning, experimenting with new tools, or proposing improvement strategies to meet the innovative capabilities expected of their future positions. Compared to self-satisfaction-oriented learning behaviors driven by basic psychological needs and intrinsic motivation, career needs offer a more purposeful and forward-looking motivational pathway—the underlying motivation often stems from a desire to enhance professional competitiveness and fulfill one’s role (Khampirat, 2021). Therefore, this motivational mechanism is more instrumental and directional, particularly evident in vocational education settings. Furthermore, research has found that career needs not only directly influence innovative behavior but may also indirectly enhance it by strengthening students’ intrinsic motivation. In other words, students’ clear understanding of and urgent need for future career development may amplify the effects of psychological need satisfaction, creating a synergistic effect at the motivational level and leading to a stable, sustained propensity for innovative behavior (Xiang et al., 2024). This finding echoes research in career motivation theory and organizational psychology on the role of goal congruence in behavioral internalization. At the practical level of education, this study highlights the critical role of curriculum design and instructional content in responding to students’ career needs. Vocational education should prioritize students’ career development expectations and strengthen the coupling between instructional content and industry development trends (Kreuzer and Weber, 2018; Messmann and Mulder, 2011). By introducing real-life situational tasks, industry cases, and interdisciplinary collaborative projects, students’ sense of career relevance to the course content can be effectively enhanced, thereby increasing their participation and innovation input.

### 4.3 The role of basic psychological needs satisfaction in innovative behavior

This study further emphasizes the critical role of satisfying students’ basic psychological needs in stimulating their innovative behavior. Results show that when students experience high levels of autonomy, competence, and social connectedness in their learning environment, they are more likely to develop strong intrinsic motivation, leading to more creative behavior in both academics and practice (Fischer et al., 2019; Saether, 2020). This finding is highly consistent with the core proposition of SDT: that satisfying basic psychological needs is a prerequisite for activating and maintaining intrinsic motivation, the core psychological mechanism driving sustained engagement and innovative behavior. In the context of vocational education, teaching practices that support basic psychological needs not only help improve students’ engagement and motivation but also significantly promote the development of their innovative abilities (Lozano-Jiménez et al., 2021; Okada, 2023; Ryan et al., 2011). Schools and teachers can address these psychological needs through various approaches, such as providing learning task choice to enhance autonomy, designing moderately challenging tasks to foster a sense of competence, and fostering a collaborative and supportive atmosphere to strengthen social connectedness. Notably, this study found that the need for autonomy has a particularly significant impact on students’ innovative behavior. When students have decision-making power and a sense of control over their learning, they are more likely to take responsibility for their learning, express their own insights, and explore alternative problem-solving approaches. As SDT points out, autonomy not only strengthens students’ sense of the value of learning but also provides them with greater psychological investment and goal alignment when facing complex tasks. Therefore, in vocational education practice, institutional and pedagogical interventions should be used to strengthen students’ sense of autonomy. For example, encouraging students to exercise subjective initiative in course selection, project theme setting, and assessment methods can effectively stimulate their learning motivation and innovative engagement. Furthermore, providing students with opportunities to make decisions and provide feedback in real-world professional scenarios will help them develop a self-perception framework that “innovation is responsibility.” In short, creating an educational environment that supports students’ independent choices, the development of their abilities, and a sense of social belonging not only strengthens their intrinsic motivation but also fundamentally enhances their creative thinking and practical abilities. This psychologically driven instructional design concept offers a viable path for the systematic cultivation of students’ innovative abilities within the vocational education system and provides empirical support for the “student-centered” approach in current educational reform.

## 5 Research limitations and future directions

Although this study reveals the significant impact of basic psychological need satisfaction, intrinsic motivation, and the internalized expression of career needs on students innovative behavior, several limitations remain, warranting further research and refinement. First, the data in this study were primarily drawn from self-report questionnaires, which may be subject to social desirability bias. Respondents may have tended to present behaviors and attitudes that align with societal and educator expectations rather than their actual subjective experiences and behavioral responses. This bias could lead to common-method bias, which may distort the true correlations among research variables. Therefore, future research could adopt diverse methodological strategies, such as behavioral observation, interviews, situational simulation experiments, or longitudinal follow-up designs, to enhance the ecological validity and explanatory power of the data. Second, the sample was limited to students from vocational education institutions in mainland China, which, to some extent, limits the cross-cultural applicability of the findings. Students’ cognitive structures, value orientations, and behavioral responses to the internalized expression of career needs may differ significantly across cultural contexts. Moreover, the cultural and institutional contexts of vocational education in China may have influenced students’ perceptions of innovation and the internalized expression of career needs, factors that should be taken into account when interpreting the results. The social expectations, institutional practices, and educational policies in China may have shaped students’ intrinsic motivation and innovative behavior differently from those in other countries. In particular, countries and regions differ in their institutional arrangements and social expectations regarding the cultural definition of “innovation” and the ways in which educational systems encourage it. Therefore, future research should conduct cross-cultural comparative studies to examine the universality and specificity of the relationship between the internalized expression of career needs and innovative behavior across different cultural contexts, thereby expanding the theoretical scope of the internationalization of vocational education. Third, career aspiration itself is a multidimensional construct encompassing aspects such as skill acquisition, career development, social status, and professional identity. However, this study did not explore this construct in detail, failing to reveal the potential differential impacts of different dimensions of career aspiration on students’ innovative behavior. Therefore, future research should systematically categorize career aspirations and develop theoretical frameworks to examine the specific pathways through which each dimension influences motivational generation and behavioral expression. For example, aspirations for professional skills may more directly stimulate technological innovation, while aspirations for social status or career advancement may drive longer-term, systemic innovation. Finally, vocational education in different disciplinary contexts has distinct knowledge structures and innovation requirements. This study did not deeply distinguish between disciplinary differences, which may limit the interpretability of the findings. Future research could further explore the moderating effect of disciplinary type on the relationship between career aspiration and innovative behavior. For example, engineering and technology students may be more inclined toward product or process innovation, while humanities students may be more focused on innovation in social services and cultural expression. Insights from disciplinary differences will help develop more targeted educational intervention strategies to promote students’ innovative capabilities across various professional contexts effectively. In summary, while this study provides theoretical and empirical support for understanding the relationship between the internalized expression of career needs and students’ innovative behavior, many issues remain to be explored in depth. Future research should focus on the multidimensional construction of the internalized expression of career needs, cross-cultural and cross-disciplinary comparisons, and the introduction of methodological diversity to promote further development in the field of vocational education, both in theoretical exploration and in practical application.

## 6 Conclusion

This study provides important theoretical and empirical support for understanding the psychological mechanisms underlying innovative behavior among vocational education students. The results indicate that intrinsic motivation and career needs are the core drivers of student innovation, while satisfaction of basic psychological needs serves as a key mediating factor in the relationship between the two. This finding further validates the applicability of SDT in vocational education and sheds light on how motivational mechanisms translate into concrete creative behaviors within educational contexts. The empirical evidence from this study suggests that vocational education institutions seeking to enhance students’ innovative capabilities systematically should prioritize aligning curriculum design with career orientation, while also strengthening support for students’ autonomy, sense of competence, and sense of connectedness. By fostering an educational environment that encourages independent exploration, enhances a sense of learning value, and fosters a sense of belonging, schools can effectively stimulate students’ intrinsic motivation and, in turn, increase their innovative engagement in learning and practice. Furthermore, career needs are not merely external drivers; they also play a guiding role in students’ professional identity construction and the generation of innovative motivation. This study suggests that educational policymakers and frontline teachers should fully integrate industry trends into their teaching practices to ensure a close alignment between educational goals and career realities, thereby enhancing students’ perception of the current significance of educational activities and their identification with their future value. As the labor market continues to evolve, innovation has become a core competency for students as they adapt to future career challenges. Therefore, the vocational education system should strive to build a learning ecosystem that supports psychological needs, stimulates intrinsic motivation, and responds to career needs, enabling students not only to acquire professional skills but also to possess the ability and willingness to innovate continuously. Future research should further refine the multidimensional structure of career needs and examine how they influence innovative behavior across different educational stages, professional orientations, and cultural contexts. This will provide a more solid theoretical foundation and practical path for improving the quality and strategic transformation of vocational education.

## Data Availability

The raw data supporting the conclusions of this article will be made available by the authors, without undue reservation.
